# The incidence of *Orientia tsutsugamushi* infection in rural South India

**DOI:** 10.1017/S0950268822001170

**Published:** 2022-06-29

**Authors:** Carol S. Devamani, John A. J. Prakash, Neal Alexander, John Stenos, Wolf-Peter Schmidt

**Affiliations:** 1 Department of Clinical Microbiology, Christian Medical College, Vellore, India; 2 MRC International Statistics and Epidemiology Group, London School of Hygiene and Tropical Medicine, London, UK; 3 Australian Rickettsial Reference Laboratory, Geelong, Australia; 4 Department for Disease Control, London School of Hygiene and Tropical Medicine, London, UK

**Keywords:** Incidence, *Orientia tsutsugamushi*, scrub typhus, serology

## Abstract

Scrub typhus is a common bacterial infection in Asia caused by *Orientia tsutsugamushi*. This serological cohort study estimated the incidence of infection in a rural population in South India. Participants were enrolled through systematic sampling in 46 villages at baseline, and revisited the following year. Blood samples were tested for IgG antibodies using ELISA, followed by indirect immunofluorescence assays (IFA) in those positive for ELISA at both rounds. A case was defined as sero-conversion (ELISA), or at least a 4-fold titre increase (IFA), between the two time points. In addition to crude incidence rate estimates, we used piecewise linear rates across calendar months, with rates proportional to the monthly incidence of local hospital cases to address seasonality and unequal follow-up times. Of 402 participants, 61.7% were female. The mean age was 46.7 years, (range 13–88). 21 participants showed evidence for serological infection. The estimated incidence was 4.4 per 100 person-years (95% CI 2.8–6.7). The piecewise linear rates approach resulted in a similar estimate of 4.6 per 100 person years (95% CI 2.9–6.9). Considering previous estimates of symptomatic scrub typhus incidence in the same study population, only about 2–5% of infections may result in clinically relevant disease.

## Background

Scrub typhus is a febrile illness endemic to much of the South Asia, East Asia and Southeast Asia regions [1], with cases also reported in Chile [2] and East Africa [3]. Severe infection is marked by acute respiratory distress syndrome (ARDS), shock, renal failure and meningo-encephalitis [4]. Annually, one million symptomatic scrub typhus cases may occur globally [5]. Scrub typhus is caused by intracellular bacteria of the genus *Orientia*, with *O. tsutsugamushi* being the dominant species in Asia [6]. A high strain diversity of *O. tsutsugamushi* has been shown to contribute to the lack of sustained immunity against re-infection [7]. *Orientiae* are transmitted by trombiculid mite larvae, also known as chiggers [8]. Mite hosts include a wide range of small mammals, in particular rodents, but also humans and birds [9]. *O. tsutsugamushi* is maintained in mites through vertical and horizontal transmission. Mite larvae do not typically become infected by feeding on mammals [8]. A human case of scrub typhus is unlikely to become a source for new infections. The risk of human infection depends on the exposure of humans to chiggers. Exposure in turn depends on environmental factors influencing chigger abundance such as humidity, temperature and vegetation [9], as well as human behavioural factors related to the risk of chigger infestation, such as agricultural activities [10, 11]. As a consequence, the infection shows a marked seasonality, in South India in the form of a gradual increase during the rainy season and a gradual decline during the hot and dry months, with little inter-annual variation [12], likely reflecting seasonal changes in chigger abundance and human behaviour [11].

The global burden of scrub typhus has been explored based on cross-sectional serological surveys [13] or on passive case detection [14]. Few studies on the incidence of symptomatic infection with *Orientia tsutsugamushi* have been published [15]. Brown and colleagues found an annual incidence of scrub typhus of 12/1000 in a population of plantation workers in Malaysia [16]. Using a population attributable fraction approach, we previously estimated the annual incidence of clinically apparent scrub typhus to be about 0.8/1000 in a rural setting in South India [17]. Serological studies showed that the infection may be very widespread [14]. Brown and colleagues found a cumulative incidence of serological infection of 14.6% over 7–8 months period in two villages in Malaysia [18]. A study on US military personnel travelling to rural areas of Laos, Vietnam and Cambodia for periods of around 4 weeks suggested a risk of *O. tsutsugamushi* IgG sero-conversion of 4% despite chemoprophylaxis for malaria using doxycycline (which is active against *Orientiae*) [19]. A similar study from Taiwan on military personnel stationed in an endemic region for 5 months found a risk of sero-conversion of 4% [20]. The proportion of infections leading to clinically apparent infection is unclear. In this study we revisited the control population of our earlier incidence study [17], to estimate the incidence of serological infection in the same communities.

## Methods

### Study design, enrolment

**Fig. 1.**
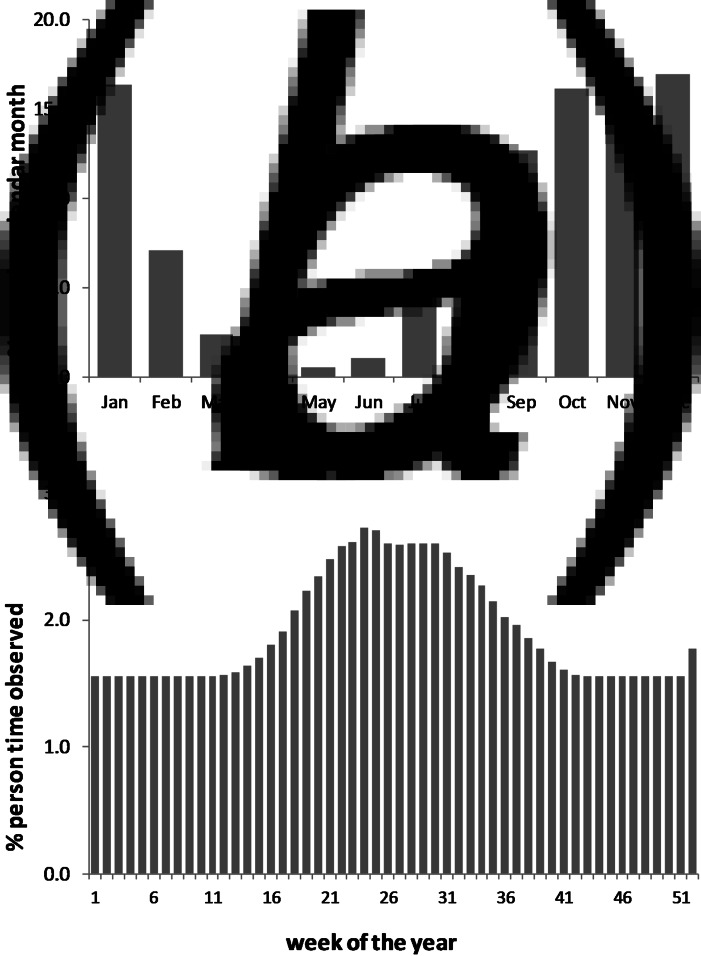
(a) Proportion of scrub typhus cases diagnosed at CMC hospital for each month of the year, averaged over the years 2006 to 2011 (extracted from [12]). (b) Distribution of observed person-time by calendar week.

Study participants of this sero-cohort were recruited from the control population of a retrospective cohort study [17]. The methods of the earlier study have been described in detail [17]. Briefly, the study was conducted in rural villages in Vellore and Ranipet Districts, Tamil Nadu (South India). Monsoon rains occur between June and December. The scrub typhus season lasts approximately from July to March with a peak from October to January (Fig. 1). Scrub typhus occurs sporadically between seasons [12].

For the original study, a total of 48 villages were enrolled. We first enrolled villages in Vellore district based on hospital admission records at the Christian Medical College Vellore (CMC). Villages were enrolled if at least two scrub typhus cases from that village were admitted to CMC during the three scrub typhus seasons from June 2014 to February 2017, resulting in 29 villages. A further 19 villages were added that were close to the 29 villages, until the intended number of 11 000 households was enrolled. In the previous study, cases were enrolled through door-to-door search aiming to cover the whole village. Controls were enrolled through systematic sampling during house-to-house screening, by selecting one household member of every 20th house at random. They were eligible for enrolment as control if they had not sought health care due to febrile illness between June 2017 and March 2018, and were living in the study area during that time. To prevent field workers from predominantly enrolling older people and females who were deemed more likely to be present, we used a stratified enrolment procedure, using the following four strata: female ≥50 years old, female *<*50 years old, male ≥50 years old, male *<*50 years old. Controls were enrolled in blocks of four, with each stratum being represented once. Controls gave a blood sample for serological testing.

562 controls were enrolled through this procedure between 23rd of March and 2^nd^ of July 2018. For the present study, the controls were visited again between 12^th^ of June 2019 and 24^th^ of October 2019 (due to funding constraints, revisiting occurred about 3–4 months later than planned). Controls were again asked to give a blood sample.

In addition to these data collected in the field, we used published data on cases of scrub typhus diagnosed at CMC [12] to calculate weighted person-time of observation by calendar week (see *Statistical analysis*).

### Laboratory analysis

Serum was separated from blood cells, divided into 3 aliquots and stored at −70 °C until testing. We used enzyme-linked immunosorbent assays (ELISA) to detect IgG antibodies to scrub typhus (Scrub Typhus Detect, InBios International, Inc., Seattle, WA, USA) following the manufacturer's specifications. This ELISA uses Karp, Kato, Gilliam and TA716 recombinant proteins of the 56-kD outer membrane protein. We used an optical density (OD) cut-off of ≥1.0 to define sero-positivity [21]. In participants ELISA IgG positive at both baseline and follow-up sample, those showing a decrease in ELISA OD of 0.5 or larger at follow up were treated as not having undergone an infection between the two time points. Those showing an OD decrease of less than 0.5, or no decrease at all were tested pair-wise using indirect immunofluorescence assays (IFA) to compare baseline and follow-up titres. We used scrub typhus IFA slides coated with three different strains of *O*. *tsutsugamushi*, namely Karp, Kato and Gilliam (Australian Rickettsial Reference Laboratory). The patients' sera (diluted initially at 1:64 in IgM serum diluent) were added to the IFA slides, and the conjugate was added to mark antigen-antibody complexes. The slides were subsequently washed, dried and observed microscopically (400 ×  magnification, Carl Zeiss MicroImaging GmbH, Göttingen, Germany). After screening at 1:64, positive sera were serially diluted using endpoint titration.

### Statistical analysis

Serological infection was defined as an individual who underwent sero-conversion or had a 4-fold or higher increase in IFA titre from baseline to follow-up to at least 1:128. Estimating the incidence of serological infection between paired samples is not straightforward in infections that are highly seasonal, unless the period of observation is exactly one year for each individual. Figure 1a shows the proportion of scrub typhus cases treated at CMC hospital for each month of the year, averaged over the years 2006 to 2011 (data extracted from Varghese and colleagues [12]), highlighting the marked seasonality of scrub typhus with peaks from September to January, and a low risk in the dry season (March to June). Most individuals in this study were observed for longer than one year, and the observation time was not equally distributed over calendar time (Fig. 1b). Observation time during low-risk months (for example April or May, Fig. 1a) may not add greatly to the risk of infection in an individual. In serological studies where the exact time point of infection is unknown, the midpoint of an individual's period under observation is often used to impute the time of infection [22]. However, in highly seasonal infections, the midpoint of the time observed in a case may not be a suitable time point to impute time of infection. External data sources may contain information on seasonal patterns which could be used to probabilistically estimate the distribution of infection time-points in a sero-cohort. In the current setting, hospital admission records of acute infection [12] comprise such a data source (Fig. 1). We estimated the annual rate first from a generalised linear model with complementary log-log link [23] ignoring seasonality. In a second approach we estimated the annual rate by maximum likelihood, taking into account the seasonal variability, as follows. We assumed a piecewise linear rate across calendar months, with the rates *λ_k_* (*k* = 1,…12) being proportional to the case numbers in Figure 1a, so that *λ_i_* *=* *w_i_λ* where *λ* is the annual rate to be estimated. For each person, the total follow-up time *t_i_* between the first and second samples was split by month, giving monthly times *t_ij_*, where *j* indexes study month (globally ranging from March 2018 to October 2019). The corresponding rates are *λ_ij_*, each of which equals one of the twelve distinct *λ_k_* values. The probability of seroconverting in a given month, conditional on seroconversion not yet having occurred, is 1-exp(−*λ_ij_t_ij_*), where exp is the exponential function, and here the index *j* = 1 corresponds to the person's first month with any follow-up. For this first month, this conditional probability equals the unconditional probability, because all people were at risk at the start of follow-up. For *j* ≥ 2, the unconditional probability of seroconverting in exactly that month is the product of (a) the conditional probability for month *j* and (b) the product over the previous months *j*’ = 1,.. *j*–1 of exp(−*λ_ij_'t_ij’_*), i.e. the probability of having seroconverted in none of the previous months. Then, the probability of seroconverting is the sum over months of the conditional probabilities. The corresponding Bernoulli deviance, i.e. −2 times the log-likelihood, was minimised as a function of the single parameter *λ* using the R function *nlminb*, and a likelihood-based confidence interval was obtained.

We used our earlier estimate of the annual incidence of clinically apparent scrub typhus leading to any health care use (0.8 per 1000 people, including purchases of medicines at local pharmacies) [17] to calculate the proportion of infections associated with clinically relevant disease.

For the risk factor analysis, we used complementary log-log models, ignoring seasonality. Exposure variables included age, sex, baseline *O. tsutsugamushi* IgG status and village-level IgG sero-prevalence at baseline in the 562 study participants enrolled as controls in the earlier study. The village-level IgG prevalence varied between 0.0% and 66.7% (mean 19.4%, median 14.6%). As in the previous study, we categorised villages as low prevalence (<15%) and high prevalence villages (≥15%,) [17]. In the model comparing the incidence rates between high prevalence and low prevalence villages, standard errors were adjusted for village-level clustering using robust standard errors.

### Ethics

The study was approved by CMC's Institutional Review Board (CMC IRB Ref: 11726) and LSHTM's Research Ethics Committee (LSHTM Ethics Ref: 16573). Written consent was obtained from all adult participants. Written or verbal assent was obtained from minors, alongside written consent from their parents/guardians.

## Results

Of 562 participants from 48 villages of the original control group, repeat samples were obtained in 402 (71.5%) from 46 villages. Of the remaining 160 individuals, 106 (18.9%) did not agree to a repeat sample, 36 (6.4%) could not be contacted and 18 (3.2%) had died.

Of the 402 participants, 248 (61.7%) were women. This proportion was 51.9% in those without repeat sample, *P* = 0.035. The mean age was 46.7 years (range 13 to 88, s.d. 14.5). This was higher than in the 160 without repeat sample (mean age 42.7, *P* = 0.016). The mean time of observation between baseline and follow up was 450 days, (range 353–547 days).

**Fig. 2.**
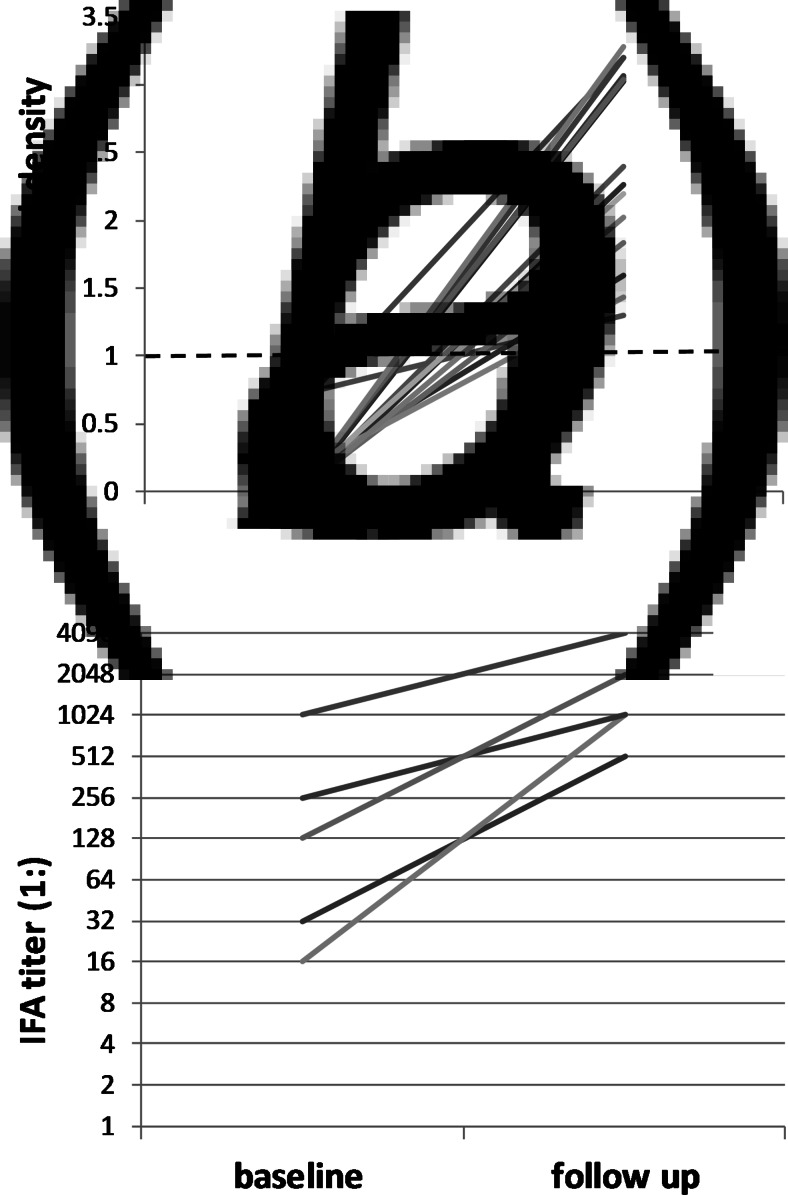
(a) Optical densities of the 16 participants negative for IgG ELISA at baseline and positive at follow up suggesting serological infection; (b) 5 cases positive for IgG ELISA at baseline and follow-up showing at least a 4-fold titre increase in IgG IFA, suggesting serological infection.

Ninety-nine (24.6%) of 402 participants were positive for IgG ELISA at baseline, 69 (17.2%) were positive at follow up. Of those positive at baseline, 53 (13.2% of 402) were positive for IgG also at follow up. Of the 303 participants negative for IgG at baseline, 16 (5.3% of 402) were found to be positive for IgG at follow up (Fig. 2a). Among those 99 positive at baseline, 46 converted to sero-negativity on ELISA (46%).

**Fig. 3.**
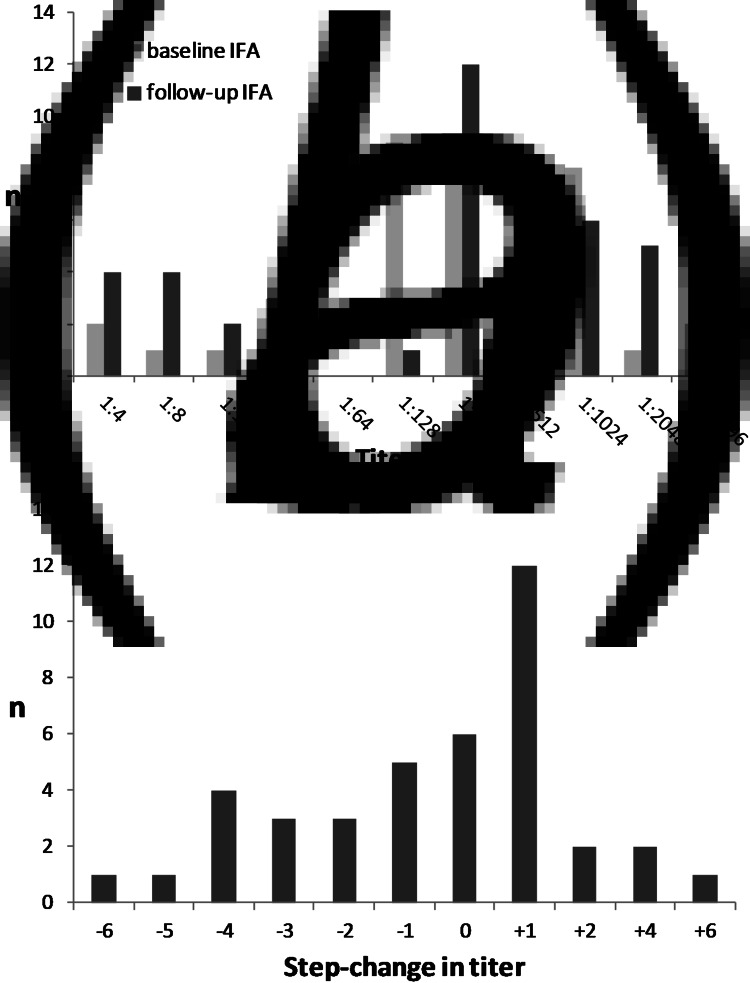
(a) Distribution of IFA titres at baseline and follow up in 40 individuals positive for ELISA at baseline and follow-up (*n* = 40); (b) Distribution of titre step-changes from baseline to follow-up in these 40 individuals.

Of the 53 participants positive for IgG at baseline and follow up, IFA was done in 40 samples, excluding those with a decrease in OD of 0.5 or higher. Of 40 sample pairs undergoing IFA, 5 individuals showed a 4-fold titre increase or higher to at least 1:128 (Fig. 2b) meeting our case definition. The distribution of titres at baseline and follow up is shown in Figure 3a. Twelve individuals had a 2-fold (one-step change) titre increase (Fig. 3b), of which the increase was to at least 1:128 in 11 individuals. Six had no change, and 17 showed a decrease. In total, we identified 21 cases – 16 based on ELISA sero-conversion and 5 based on IFA titre increase.

The total number of person-years of observation (PYO) was 495.3. The crude incidence rate (complementary log-log model) ignoring seasonality was 4.4 per 100 person years, (95% CI 2.8–6.7). The estimated incidence derived from assuming a piecewise linear rate across calendar months proportional to hospital admission data was 4.6 per 100 person-years (95% CI 2.9–6.9).

Women were found to have a 30% higher rate of serological infection compared to men, but the confidence interval was wide and included 1 (Table 1). There was no linear association between age and incidence rate. Those aged above 50 years tended to have a lower risk of serological infection compared to younger age groups, but statistical support was low. Baseline IgG status did not affect incidence.

**Table 1.** Risk factor analysis

	*N*	PYO^a^	Cases	Rate ratio	95% CI
Overall	402	447	21	–	–
Sex					
Male	154	172	7	1 (ref)	–
Female	248	275	14	1.3	0.5, 3.1
Age (per 10 years increase)	402	447	21	1.0	0.7, 1.3
Age group (years)					
13–25	64	71	3	1.1	0.3, 4.3
26–50	202	226	12	1.4	0.5, 3.6
51–88	136	150	6	1 (ref)	–
IgG ELISA baseline status					
Negative^b^	303	343	16	1 (ref)	–
Positive^c^	99	104	5	1.0	0.4, 2.7
Village-level sero-prevalence					
<15%	195	237	4	1 (ref)	–
≥15%	207	210	17	4.5	1.7, 12.0

a PYO- person-years of observation.

b serological infection defined based on ELISA sero-conversion.

c serological infection defined based on IFA titre increase.

Participants living in villages with a sero-prevalence of 15% or higher had a 4.8 times higher rate of infection as compared to those in villages with less than 15% sero-prevalence. A fever history during the observation period was reported by only 3 individuals, none of which showed evidence for serological infection.

Accounting for incidence data collected previously (see methods) suggests that only about 1.8% (0.0008/0.044) of *O. tstsutusgamushi* infections results in a clinically relevant infection.

## Discussion

In this cohort study conducted in rural villages in South India, we found a high incidence of serological infection with *O. tsutsugamushi*, the causative agent of scrub typhus, annually affecting almost one in 20 study participants living in this setting. Most of these infections appear to have been asymptomatic or associated with minimal symptoms.

Our study suggests that possibly less than 2% of infections may lead to clinically relevant infection. However, in the previous study we found evidence for under-reporting of fever episodes of about 50%, implying that, more plausibly, about 3%–5% of infections may result in a clinical disease leading to any health care use. The latter included purchase of medicines at local pharmacies which is a common practice in the area in any case of fever.

One potential limitation is that study villages were selected for presumed endemicity for scrub typhus and do not represent a random sample. Generalising the estimates to other settings is therefore not easy. Similarly, our incidence estimate is difficult to compare with the cumulative incidence of 14.6% over a 7–8 months period in two highly endemic villages in Malaysia identified by Brown and colleagues [18].

Our finding of most infections with *O. tsutsugamushi* being asymptomatic or associated with minimal symptoms stands in contrast to the high clinical attack rates of the disease observed in populations temporarily visiting endemic areas, such as military personnel [24, 25] or plantation workers [9]. For example, three out of a group of 6 Italian travellers to Laos developed clinical scrub typhus after a 12-day hiking trip [26]. Olson and Bourgois reported a proportion of 30% of infections resulting in clinical symptoms among military personnel temporarily stationed in a highly endemic region [20]. Regional strains of *O. tsutsugamushi* may well differ with regard to pathogenicity [9]. Another explanation may be that these groups were immune-naïve to the infection and therefore at a higher risk of developing clinical disease than those living in endemic areas [9]. However, one would then expect the risk of clinical scrub typhus to decrease with age in endemic areas, which has not been observed in our setting in South India [27]. In particular, the risk of severe scrub typhus appears to be particularly high in older people, with IgG sero-positivity suggestive of prior infection not protecting from severe infection [27]. Alternatively, high clinical attack rates in selected groups of people residing at a single location may also be due to a spatial and temporal clustering of mite larvae carrying a particularly pathogenic strain of *O. tsutsugamushi*. Mites are typically assumed to occur in distinct ‘mite islands’ that may cause localised outbreaks [9]. Within such mite islands, there may perhaps also be clusters of pathogenic *O. tsutsugamushi* strains, causing local outbreaks of clinical disease within a wider area where asymptomatic infections, on average, predominate. This could reconcile the contrasting findings from our study and the above-mentioned distinct outbreaks with high clinical attack rates. However, these may also have erroneously resulted from publication bias favouring conspicuous outbreaks. Epidemiological studies comparing spatial clustering of serological infection and clinically apparent infection could help to shed further light on this. Such studies should use active case finding at shorter intervals to minimise under-reporting of fever episodes.

When estimating incidence from observation periods which are staggered over time, statistical methods should incorporate seasonality, which can be marked for some infections including scrub typhus. We addressed this seasonality by using external data in the form of local hospital records of scrub typhus cases to calculate the approximate risk of infection in the population by calendar month. These data allowed estimating linear piecewise rates by calendar month. The resulting point estimate was only slightly higher than the crude estimate from the complementary log-log model, suggesting that in this case, seasonality had only limited impact on the analysis.

Given the large variation in the burden of scrub typhus across different settings [14], obtaining precise regional data on scrub typhus incidence may be highly beneficial for local health systems. The statistical method proposed here may facilitate epidemiological studies by allowing more flexibility in the timing of blood sample collection. The approach may be applicable to other seasonal infections, and also to highly irregular epidemic curves as observed in the SARS-CoV-2 pandemic [28].

The high proportion of conversion from IgG sero-positivity to sero-negativity and the associated decrease in IgG sero-prevalence from the baseline to the follow up survey from 25% to 17% was unexpected. In the same study population, IgG sero-prevalence strongly increases with age [17], which should have led to a slight increase in the prevalence over one year. One explanation for the decrease in sero-positivity may be a recent substantial decrease in scrub typhus incidence in the study area. Since the end of the present study, we have been conducting a population-based cohort study linked to hospital admissions in the study area [29], and in interim analyses have not identified any such decrease in incidence. Further, the delayed second round of sampling to a time of year just before the marked autumn increase in scrub typhus may have contributed to the observed decrease in sero-prevalence by allowing for more time to convert to sero-negativity. The longevity of IgG antibodies against O. tsutsugamushi following infection has been found to be between one to over two years [21, 30–32], depending on whether infection is primary or recurrent [21, 30]. As suggested by Saunders and colleagues [33], data on the incidence of scrub typhus infection and conversion from sero-positivity to sero-negativity could inform model-based approaches to estimate the incidence of infection in settings with limited data availability. The strong association between sero-prevalence and incidence in our study (Table 1) indicates that by using adequately parameterised models, sero-prevalence data (which are more widely available than incidence data [14]) may be suitable to predict incidence across settings. Differences in pathogenicity among *O. tsutsugamushi* strains may complicate such efforts.

The high proportion of non-response of around 28% at the follow-up visit is a concern and may have affected the incidence estimate by biasing the sample towards certain socio-demographic characteristics. Due to logistical constraints we did not collect extensive socio-demographic data which would have allowed exploring the potential of participants dropping out of the study to impact on the findings. Participants were recruited through stratified systematic sampling to favour balanced inclusion of age groups and genders, but very young ages were under-represented. The sample was skewed towards older people and women, who were more likely to be present at both visits. Age and sex, however, were not strongly associated with infection.

The relatively small sample size of 402 participants resulted in only 21 cases. The resulting confidence interval of the crude point estimate of 4.4 (3.0. 7.4) in our view indicates reasonable precision. However, larger studies will be needed to estimate incidence in subgroups and conduct risk factor analyses.

Our main method of testing was ELISA, which has a higher specificity than IFA in our setting [34]. We used IFA to determine sero-infection in those ELISA positive at both time points, screening out those likely to not having undergone infection based on ELISA.

To conclude, this study found a high risk of infection with *O. tsutsugamushi* in this highly endemic region. Our data suggest that only a small fraction of these infections may lead to clinically relevant disease.

## Data Availability

The data underlying this article will be shared on reasonable request to the corresponding author, subject to approval by the IRB committee of the Christian Medical College, Vellore.

## Supplementary material

For supplementary material accompanying this paper visit http://doi.org/10.1017/S0950268822001170. click here to view supplementary material

## References

[1] Kelly DJ (2009) Scrub typhus: the geographic distribution of phenotypic and genotypic variants of *Orientia tsutsugamushi*. Clinical Infectious Diseases 48(suppl. 3), S203–S230. 19220144 10.1086/596576

[2] Weitzel T (2016) Endemic scrub typhus in South America. New England Journal of Medicine 375, 954–961. 27602667 10.1056/NEJMoa1603657

[3] Maina AN (2016) Q Fever, scrub typhus, and Rickettsial diseases in children, Kenya, 2011–2012. Emerging Infectious Diseases 22, 883–886. 27088502 10.3201/eid2205.150953 PMC4861507

[4] Abhilash KP (2016) Acute undifferentiated febrile illness in patients presenting to a tertiary care hospital in South India: clinical spectrum and outcome. Journal of Global Infectious Diseases 8, 147–154. 27942194 10.4103/0974-777X.192966 PMC5126753

[5] Watt G and Parola P (2003) Scrub typhus and tropical rickettsioses. Current Opinion in Infectious Diseases 16, 429–436. 14501995 10.1097/00001432-200310000-00009

[6] Tamura A (1995) Classification of Rickettsia tsutsugamushi in a new genus, Orientia gen. nov., as *Orientia tsutsugamushi* comb. nov. International Journal of Systematic Bacteriology 45, 589–591. 8590688 10.1099/00207713-45-3-589

[7] Smadel JE (1950) Immunity in scrub typhus: resistance to induced reinfection. AMA Archives of Pathology 50, 847–861. 14789327

[8] Paris DH (2013) Unresolved problems related to scrub typhus: a seriously neglected life-threatening disease. American Journal of Tropical Medicine and Hygiene 89, 301–307. 23926142 10.4269/ajtmh.13-0064 PMC3741252

[9] Elliott I (2019) Scrub typhus ecology: a systematic review of Orientia in vectors and hosts. Parasites and Vectors 12, 513. 31685019 10.1186/s13071-019-3751-x PMC6829833

[10] Devamani CS (2020) Risk factors for scrub typhus, murine typhus, and spotted fever seropositivity in urban areas, rural plains, and Peri-forest hill villages in South India: a cross-sectional study. American Journal of Tropical Medicine and Hygiene 103, 238–248. 32458785 10.4269/ajtmh.19-0642 PMC7356468

[11] Elliott I (2021) *Orientia tsutsugamushi* dynamics in vectors and hosts: ecology and risk factors for foci of scrub typhus transmission in northern Thailand. Parasites and Vectors 14, 540. 34663445 10.1186/s13071-021-05042-4 PMC8524837

[12] Varghese GM (2016) Epidemiology & risk factors of scrub typhus in South India. Indian Journal of Medical Research 144, 76–81. 27834329 10.4103/0971-5916.193292 PMC5116902

[13] Xu G (2017) A review of the global epidemiology of scrub typhus. PLoS Neglected Tropical Diseases 11, e0006062. 29099844 10.1371/journal.pntd.0006062 PMC5687757

[14] Bonell A (2017) Estimating the burden of scrub typhus: a systematic review. PLoS Neglected Tropical Diseases 11, e0005838. 28945755 10.1371/journal.pntd.0005838 PMC5634655

[15] Salje J (2021) Rickettsial infections: a blind spot in our view of neglected tropical diseases. PLoS Neglected Tropical Diseases 15, e0009353. 33983936 10.1371/journal.pntd.0009353 PMC8118261

[16] Brown GW (1976) Scrub typhus: a common cause of illness in indigenous populations. Transactions of the Royal Society of Tropical Medicine and Hygiene 70, 444–448. 402722 10.1016/0035-9203(76)90127-9

[17] Devamani CS (2019) Hospitalisations and outpatient visits for undifferentiated fever attributable to scrub typhus in rural South India: retrospective cohort and nested case-control study. PLoS Neglected Tropical Diseases 13, e0007160. 30802243 10.1371/journal.pntd.0007160 PMC6405239

[18] Brown GW, Robinson DM and Huxsoll DL (1978) Serological evidence for a high incidence of transmission of Rickettsia tsutsugamushi in two Orang Asli settlements in Peninsular Malaysia. American Journal of Tropical Medicine and Hygiene 27, 121–123. 415625 10.4269/ajtmh.1978.27.121

[19] Corwin A (1999) Scrub typhus and military operations in Indochina. Clinical Infectious Diseases 29, 940–941. 10589920 10.1086/520468

[20] Olson JG and Bourgeois AL (1977) Rickettsia tsutsugamushi infection and scrub typhus incidence among Chinese military personnel in the Pescadores Islands. American Journal of Epidemiology 106, 172–175. 888820 10.1093/oxfordjournals.aje.a112448

[21] Schmidt WP (2019) Antibody response following scrub typhus infection: clinical cohort study. Tropical Medicine and International Health 24, 1455–1464. 31660667 10.1111/tmi.13322

[22] Vandormael A (2020) HIV incidence declines in a rural South African population: a G-imputation approach for inference. BMC Public Health 20, 1205. 32762668 10.1186/s12889-020-09193-4 PMC7409400

[23] Martuzzi M and Elliott P (1998) Estimating the incidence rate ratio in cross-sectional studies using a simple alternative to logistic regression. Annals of Epidemiology 8, 52–55. 9465994 10.1016/s1047-2797(97)00106-3

[24] Sayers MH and Hill IG (1948) The occurrence and identification of the typhus group of fevers in Southeast Asia command. Journal of the Royal Army Medical Corps 90, 6–22. 18903197

[25] Griffiths JT Jr (1947) A further account of tsutsugamushi fever at Sansapor, Dutch New Guinea. Journal of Parasitology 33, 367–373. 20256989

[26] Costa C (2021) Imported scrub typhus in Europe: report of three cases and a literature review. Travel Medicine and Infectious Disease 42, 102062. 33862243 10.1016/j.tmaid.2021.102062

[27] Devamani CS (2021) High initial IgG antibody levels against *Orientia tsutsugamushi* are associated with an increased risk of severe scrub typhus infection. PLoS Neglected Tropical Diseases 15, e0009283. 33735183 10.1371/journal.pntd.0009283 PMC8009433

[28] Sabino EC (2021) Resurgence of COVID-19 in Manaus, Brazil, despite high seroprevalence. Lancet 397, 452–455. 33515491 10.1016/S0140-6736(21)00183-5 PMC7906746

[29] Devamani CS (2020) The Epidemiology of Rickettsial Infections in South India: Cohort Study. Available at https://clinicaltrials.gov/ct2/show/NCT04506944.

[30] Bourgeois AL (1982) Humoral and cellular responses in scrub typhus patients reflecting primary infection and reinfection with Rickettsia tsutsugamushi. American Journal of Tropical Medicine and Hygiene 31, 532–540. 6805348 10.4269/ajtmh.1982.31.532

[31] Ha NY (2017) Longevity of antibody and T-cell responses against outer membrane antigens of *Orientia tsutsugamushi* in scrub typhus patients. Emerging Microbes and Infections 6, e116. 29259327 10.1038/emi.2017.106 PMC5750460

[32] Varghese GM (2018) Kinetics of IgM and IgG antibodies after scrub typhus infection and the clinical implications. International Journal of Infectious Diseases 71, 53–55. 29653201 10.1016/j.ijid.2018.03.018 PMC5985369

[33] Saunders JP (1980) The longevity of antibody to Rickettsia tsutsugamushi in patients with confirmed scrub typhus. Transactions of the Royal Society and Tropical Medicine and Hygiene 74, 253–257. 10.1016/0035-9203(80)90254-0 6770503

[34] Kannan K (2020) Performance of molecular and serologic tests for the diagnosis of scrub typhus. PLoS Neglected Tropical Diseases 14, e0008747. 33180784 10.1371/journal.pntd.0008747 PMC7660479

